# Chapman’s Introductory Lecture

**Published:** 1856-12

**Authors:** C. B. Chapman


### AN INTRODUCTORY LECTURE,

*To the Class of the Ohio College of Dental Surgery;—Session of* 1856–7.

BY C. B. CHAPMAN, A. M., M. D.

[Published by request of the class.]

GENTLEMEN: I have chosen as the subject of this my introductory, to the course of lectures on which we are now entering, the origin and history of institutions of learning, particularly, those for instruction in science, in different periods of historic record. Every period possesses something interesting to add to the common whole and will be found to present something on which we may with profit to ourselves indulge the impulse of gratitude. Every thought, every impulse—every principle or rule of action, as well as every living thing, has originated in a germ, or its type. The being who rules in the heavens and among the inhabitants of this lower sphere, has in but few instances brought anything at once into mature existence. To be born—to grow—to arrive at maturity—to die, and to decay, is but the history of every living thing, as well as every work of creation. Institutions of learning have been progressive in their character, from those of ancient *Greece* and *Rome* down to the present time. In the early periods of learning in those countries, the youth of a nation were assembled, where they listened to oral teachings in groves and other convenient places. The grove and the market place, not affording the necessary protection at all seasons, led to the erection of suitable buildings for the accommodation of those vast assemblages. This led to some of the most approved orders of architecture now in use. The *Piazza* and the *Portico* were the earliest names by which these structures were designated. The Academy, the University, and the College are organizations for instruction, of comparatively modern origin, and the history of the origin of each will be referred to in its appropriate place. A new arrangement of place was demanded on the change of plan of instruction from oral to the written method, or where a combination of the two prevailed. When the more exact method of written instruction was introduced, it necessarily led to a limitation of numbers, and exactness of time, when instruction in a given department was imparted. The early teachers had no bounds set to their field of instruction, but were permitted to wander wherever fancy might lead them, throughout the whole domains of learning. The university admits of a different construction in the various countries where they exist, inasmuch as the genius of every nation must impart something of its own peculiarities into its educational establishments. In no country does the university cover so wide a range as in Germany, although their origin may be traced to the foundation of the universities of Oxford and Cambridge.

These places seem to have, rather incidentally afforded some attractions to students, who congregated in the early centuries at these places, and pursued their studies under such teachers as they severally chose to employ. As some students sought those places whose means were inadequate to meet the expenses incident to their wants, eventually led some benevolent persons to contribute for the support of such classes of scholars as they saw fit to select, and this naturally led them to select teachers for their beneficiaries. This afterward led to community of interest, and eventually the whole community became associated to listen to the instructions of favorite lecturers.

In these universities some peculiar privileges are still enjoyed. The Chancellor is but an honorary office in the main, and is held by some person in the realm who is rarely in attendance, and performs no labor as instructor. The Vice-Chancellor not only presides at the university, but also performs the duties of magistrate, which duty he often executes on delinquent students in a summary manner.

Degrees were at first but licenses to teach but were afterward sought on account of the honor which they conferred.

Among the Greeks we find a description of the first schools in which *Reading*, *Writing*, *Arithmetic*, *Poetry*, *War* and *Gymnastics* were taught. Thales, Anaxagoras, Hermelites and

Pythagoras, were really instructors like those in modern universities of the highest order, devoting their lives to the investigation of truth in science, to the advancement of knowledge by the public instruction of eager listeners who gathered around them. These were followed by Socrates at Athens, then the centre of scientific culture—in the groves of Academus and by Aristotle in the Lyceum. After having been subdued by the Romans, that of Athens remained for centuries afterward the most celebrated high school in the world, where the most celebrated Romans of the republican period, as *Cicero*, *Cæsar*, *Cato* and *Brutus* advanced in the studies peculiar to that period, where the life of a student in which customs prevailed that would not render such life a smooth one in the estimation of modern students, with whom clubs and races prevail.

In the 11th and 12th centuries men appeared who drew around them crowds of disciples to listen to the teachings of science till then unknown in the convents and collegiate schools. These were mostly laymen, and independent of the monastic orders of the day. The high schools that thus arose were superceded by universities, a term that seems originally to have meant a community, association, or incorporation, and not a school for the whole range of instruction as now understood. At this period *Rhetoric*, *Dialectics* and *Theology* were taught at the Sorbonne in Paris, by Champeau and Abelard. The Roman law was taught at Bologna, and instruction in medicine was principally concentrated at Salermo, It was left to a more enlightened period, to place the sciences upon which medicine is founded in their true relations. For the accomplishment of this object time was demanded, and although medicine was at this time mainly an empirical practice, we are indebted to an age anterior to the Christian era for the record and promulgation of some principles or facts in medical science, which will ever remain as indelible as the most conspicuous of nature's own works. To Hippocrates are we indebted as the first who observed and promulgated the laws that govern the occurrence of the febrile paroxysm. He flourished about 460 years before Christ, acquired a high reputation among his countrymen, which has even descended to our day ; and his opinions were long respected as oracles not only by the schools of medicine but also in the courts of law. He was not content with the empirical knowledge and practice which he derived from his cotemporaries and predecessors, but observed and judged carefully for himself, and adopted those principles only, which seemed founded upon sound reasons, upon truths susceptible of demonstration. This, gentlemen, is the only method by which science can truly be advanced. Centuries of time have been more than lost to the medical as well as the general scientific world, by relying upon the fabulous productions of the early ages. Medicine was long regarded as a mysterious art, and those who engaged in its solemn rites, were regarded with the awe which was more naturally bestowed upon those whom they supposed, held communion with the hidden things of other spheres. To Galen are we indebted for his earnest labors in searching out such articles from the vegetable kingdom as were useful in the cure of maladies. He made an imperfect compendium of botany which was greatly enriched during the 16th century by the writings of Linnæus, whose classification is still respected by many who cultivate that science. To Aristotle are we indebted for some of the early lessons in physiology and the principles of pathology. He was born 384 years before Christ. He followed his studies with such diligence as to surpass all of Plato's school. He greatly restricted himself in his food and rest, and that he might guard himself from excess in the latter, used to lie with one hand out of bed with a brass ball in it which by falling into a basin of the same metal would awake him. It would seem that medicine was not pursued as a distinct science in his time, for although he may be justly referred to as one of the fathers of medicine, he was employed by Phillip of Macedon, as tutor for his son Alexander, and during three years devoted much of his time in instructing the Royal youth in *Politics*, *Natural Philosophy*, *Ethics* and *Rhetoric*, and according to Plutarch, in a certain kind of *Philosophy*, which he taught no one else. The departments of medical science to which Aristotle devoted his best energies were destined to make but small advances untill the early part of the last century, when after thirty-six years of unremitting toil, upon the shores of the Lake of Geneva, in the city of Lausanne, was produced a system of Physiological and Pathological science founded upon actual, and truthful observations of the phenomena that govern the growth, sustenance and decay of living beings. It was at Lausanne, and in 1757 that Haller published, after many years of unremitting labor, his remarkable work on Physiology—Elementia Physiologia Corpus Humani.

A little less than one century ago, in the midst of those rugged mountains, in one of the by-ways of Continental Europe, was laid the foundation of that Physiological and Pathological structure, upon which Bichat and his cotemporaries and successors reared an enduring structure, because its materials are truths sought out in the manifestations of nature.

In 1851 I had the satisfaction of being at Hotel Dieu, in Paris, when M. Roux paid a just and feeling tribute to the memory of his early patron and friend, on taking leave of his wards for his usual annual vacation, which he intimated would be principally employed in preparing a eulogy on Bichat. It was at Clamart, under the shade of its stately trees, that Bichat gave his early lessons to eager pupils who followed him thither from the halls from whence he had been driven by the jealousy of his cotemporaries. It was at Clamart that he labored to good effect in the reform of pathological science, and a fit place did this seem for his mortal part to repose.

Follow gentlemen, I pray you in all your pursuits in science, in the footsteps of these illustrious predecessors. The book of nature which they so diligently studied is ever open before you, and allow me to give you the strongest assurance that this book will never deceive you. If deceived you are, the fault is in your own unskilfulness as interpreters.

The origin of American colleges may be traced to an apparent deficiency of suitably educated persons to fill the professions of theology, law and medicine, and the posts of public trust connected with State affairs. These institutions are limited in their design, to the diffusion of learning already in existence, and not to contribute except incidentally to a further advancement in knowledge. This is true of our colleges and most of our institutions of learning, but a grade of associations exist, both in this country and the old world, which are designed to subserve a higher purpose. It is the aim of these associations to secure an advance in science, art and literature. The most efficient of these is the Institute of France. In 1793, the National Convention abolished all literary and scientific societies denominated academies, and two years after founded the Institute, the object of which was to replace the former academies. The Institue was first divided into three sections, which have been advanced to five. In order to give a concise view of this establishment, it will be necessary to refer to each division separately.

1st. The Academy of France consists of 40 members and is specially charged with the composition of the Dictionary, and extension and purification of the language. It is entrusted with the disposal of several prizes, among which are one for Poetry or Eloquence of 2,000 francs; one for the most useful work of public morals; another for some distinguished act of virtue, displayed by some poor native of France, one of 10,000 francs for the most eloquent work on French history, and every alternate year awards a gratuity of 1,500 francs to some deserving but indigent young man of letters.

2nd. The Academy of inscriptions and Belle Lettres, has for the object of its researches, the learned languages, antiquities and monuments. Their attention is particularly directed to the translation of *Greek*, *Latin*, and *Oriental* works into the French language, and the formation of Archœological collections. This Academy adjudges an annual prize of 2,000 francs for the most learned work on French history, and three medals of 500 francs each for the best work on French Antiquities.

3d. The Academy of sciences contains 65 members including 2 secretaries, 10 free academicians, besides 8 foreign associates and correspondents. This academy is divided into 11 sections, which are as follows: Geometry, Mechanics, Astronomy, Geography and Navigation, General Natural Philosophy, Chemistry, Mineralogy, Botany, Rural Economy, and Veterinary art, Anatomy and Zoology, Medicine and Surgery. This Academy adjudges a prize of 3,000 francs for Physical sciences—the same for statistics—for experimental Physiology and Mechanics. It also adjudges prizes for improvements in Medicines and Surgery, for improvements relative to the treatment of patients, for the means of rendering the arts of trade less insalubrious, and one yearly prize of ———, which was founded by the widow of *M. de la Place* the astronomer, for the most meritorious pupil of the year in the Ecole Polytecnic.

4th. The Academy of fine arts, is composed of 41 members, besides 10 free academicians and associates, it is divided into five sections which are Painting, Sculpture, Architecture, Engineering, and Musical composition. It also distributes annual prizes for the best works of students in the arts, and those who excel are sent to the French Academy at Rome, and educated at the expense of the state.

5th. The Academy of moral science and politics which was restored by order of Louis Phillipe in 1832, is divided into 5 sections which are constituted as follows: Philosophy, Moral Philosophy, Legislation, Public right and Jurisprudence Political economy and statistics History and Philosophy of history. This Academy has five associates among whom were Lord Brougham, Mr. Hallam, and McCulloch.

The Society of France, of which this is a successor, was the Royal Academy of Sciences. It was founded in 1666, by Louis XIV. His design was to perfect the arts and sciences by uninterrupted researches, to publish discoveries by correspondence with learned foreign societies. The ruling monarch intrusted a favorite minister to form a society of men, distinguished for learning and talent, who should meet under the royal protection, and communicate their respective discoveries. They were chosen from among the most celebrated in *Physics*, *Mathematics*, *History* and *Belles Lettres*.

The Royal Society of London was founded by Royal charter in 1663, though its existence dates from 1645. It was at first a voluntary association of a small number of literary men, who thus came together from being engaged in similar pursuits. In order to meet the expense of publishing its transactions, each member was required to pay £10 on his entrance, and an annual subscription of £4. A large *library*, a museum of natural history, and a collection of apparatus belongs to the society. It has published a volume of transactions each year, since 1800. From 1665 to 1800, it had published 90 volumes, though many of them were of small size in comparison with the voluminous collections since 1800. There is a donation fund for assisting scientific men in their researches, and three medals are annually adjudged, which are known as the Rumford medal, and two Royal medals. The Royal Societies of Edinburgh and Dublin are similar in their organization and aims.

It may not be out of place to refer to some of the more prominent societies of this kind in other countries, to which the term Academy is generally prefixed, and is thus used to denote a society of learned men associated for the purpose of advancing the arts and sciences, and communicating to the world the discoveries made either by their own members or by other individuals. The most distinguished German association of this kind is the Royal Academy of Sciences of Berlin, founded by Frederick the First, after the model of the Royal society of London. The Royal Spanish Academy was founded at Madrid in 1714. The Royal Academy of Sciences at Lisbon in 1779, which is after the model of that of France, by division into sections. The Royal Academy at St. Petersburgh was founded by PETER THE GREAT, and carried out by his successor, Catherine. That of Stockholm had its origin from an association of six private persons, of whom Linnæus, the celebrated naturalist is one. It cannot be denied, that the most perfect Academic model of our time is the Institute of France, which has already been elaborately described.

There is a society in which the American scholar may indulge a laudable pride, not fostered by kings or Royal charter, but the work of men engaged in the stern business of laying the foundation of this Republic.

The American Philosophical society is supposed to have originated in the association of a small number of persons of scientific tastes. It began early in the last century, at Philadelphia, as a secret club for the discussion of scientific questions. This club was called the Junto, and its number was limited to twelve. The rule requiring secrecy was declared by Franklin (who was one of its originators) to be the prevention of applications for membership from improper persons.

Another rule was the assent of candidates to a few questions, without which none were admitted. The character of these questions may be inferred from the following.

Do you love truth for truth's sake, and will you endeavor to find and receive it yourself, and communicate it to others? A proposition was once submitted to the Junto for increasing its members, which was responded to by authorizing each member to establish a like club, without imparting a knowledge of the existence of the parent society. There is no record of more than three having been the fruit of this arrangement.

The Junto did not at first claim to be a scientific body.

But the then British Provinces were fast advancing in population and prosperity, and the active mind of Franklin, ever seeking to do good, and especially anxious for the promotion of knowledge, became impressed with the importance of establishing a national Institution for the cultivation of science. He accordingly issued and distributed a printed circular, urging the formation of such a society. This proposal was the true origin of the Philosophical Society, and bears date of May 14th, 1743. The society was to be formed of ingenious men, residing in the several colonies, at least seven of whom were to be residents of Philadelphia. They were to maintain constant correspondence with other societies, as well as with their own members at a distance. The members at Philadelphia were to meet once a month, or oftener, at their own expense, to communicate to each other their observations and experiments, to receive and consider such *letters*, *communications* or *questions* as shall be sent from distant members, and direct the distribution of copies of communications that were deemed valuable, in order to procure their sentiments thereon. This circular also proposed that correspondence should be kept up with the Royal societies of London, Dublin, and Edinburgh. In order to secure division of labor, and thus more perfect investigations, to each member in Philadelphia was assigned a single department. The following is the programme under this arrangement for 1744. Dr. Thomas Bond, as Physician; Mr. John Bertram, as Botanist; Mr. Thomas Godfrey, as Mathematician; Samuel Rhodes, as Mechanician; Wm. Parsons, as Geographer; Dr. Phineas Bond, as General Natural Philosopher.

Thos. Hopkinson was President, Wm. Coleman Treasurer, and Benj. Franklin, secretary. The first president, (Thos. Hopkinson) is said to have possessed a fine genius and finished education, having been a student at Oxford. He came to this country while young, and died at the age of 42. Being fond of the pursuits of science, as well as letters, he often assisted Franklin in his electrical and philosophical experiments. Dr. Franklin thus refers to him in one of his letters, "The power of throwing off the electrical fire was first communicated to me by my ingenious friend Thos. Hopkinson, since deceased, whose virtues and integrity in every station of his life, public or private, will ever make his memory dear to those who knew him, and knew how to value him." Drs. Thos. and Phineas Bond, were both eminent and learned men. The former of them was the projector of the Pennsylvania Hospital, though he failed to establish it, until he had recourse to the sagacious and powerful influence of Franklin.

JOHN BERTRAM was the founder of the Botanic garden of Philadelphia, which still exists. But a small number of the early papers of the society were preserved.

In the minutes for 1760, the following record is found, "Charles Thomson read to the company a declamation in which from matters of fact, joined with probable conjecture, it was endeavored to account for the gossamer, or those filmy threads which are sometimes seen to cover whole fields in a dewy morning, especially in the autumn; and to show that they might be the threads of webs of the flying spider, weighed down by the particles of water they collect from the condensing of vapors upon the sun's withdrawing his beams."

The author of this paper was one of the leading spirits of the day. To act up to the pretensions of a society that took the broad name of American was no easy task.

A failure to meet such expectations was painfully felt by many of its members, and by none more keenly than by the most zealous of them all, Chas. Thomson. Many schemes were proposed from time to time, for placing the society in the position to which it aspired. At this time a paper was prepared and presented to the society by Mr. Thomson, which proposed a considerable extension of its objects. Several departments of knowledge seem to have been touched upon, but special reference was always had to immediate utility, and agriculture was a favorite topic. The paper concludes in the following words, "The spirit of enquiry is awake, and nothing seems wanting but a public society, such as the American Society is now proposed to be, formed on a plan to encourage and direct enquiries and experiments, collect and digest discoveries and inventions, and unite the labors of many to attain one grand end; the advancement of useful knowledge and improvement of our country. As Philadelphia is the centre of the colonies, as her inhabitants are remarkable for encouraging useful undertakings, why should we hesitate to enlarge the plan of our society, call to our assistance men of learning and ingenuity from every quarter, and unite in one general noble attempt, not only to promote the interests of our country, but to raise her to some eminence in the rank of polite and learned nations." These proposals were printed and distributed at the time, and were subsequently embraced in the preface to the first volume of transactions.

This year seems to have proved one of unusual activity. Large additions were made to their list of fellows and correspondents, among whom was Franklin, then in England, and other men distinguished in science. Their proceedings were no longer those of a debating club, but of a learned society. Papers were read, some of which were introduced into the printed transactions, communications were received from foreign correspondents, premiums were awarded, donations received, and a cabinet formed. On the 22d of March in this year (1768), the first scientific communication was made to the society, which stands first in the series of transactions. It is entitled, "A description of a new Orrery, planned and now nearly finished, by David Rittenhouse, A. M." This instrument has been a scientific wonder for more than three-quarters of a century. One was finished for Princeton college, and another for the college in Philadelphia.

Perhaps no more important incident has occurred in the history of the society than the observation of the transit of

Venus, taken under its direction in 1769. The importance attached to observations of this phenomenon may be inferred from the fact that Capt. Cook's first voyage was undertaken by order of the British government for the special object of carrying out astronomers to observe the transit at the island of Otaheite.

The attention of this society was first called, formally to the subject by Rev. John Ewing, a pastor of one of the Presbyterian churches of Philadelphia, then one of its secretaries, and afterward Provost of the University. By calculations carefully made by himself, he found that the beginning and a greater part of it would be visible at Philadelphia, provided the weather was favorable. He then called the attention of the society to the importance of making provision for multiplying observations of it, in different parts of the world. The wisdom of that suggestion was proved by the observation having been prevented in the north of Europe by cloud-cast skies. He then urges the society to make ample provision for the observation in Philadelphia, which he urged the more as another opportunity would not occur for more than a century. The society from this recommendation, undertook the task of providing for ample observations at Norriston, Philadelphia, and Cape Henlopen. It was found that this noble and difficult undertaking could not be sufficiently provided for from their own treasury. Accordingly a memorial was made to the Legislature, which was promptly responded to by appropriating £100 for erecting observatories and for the purchase of instruments. Committees were appointed by the society to conduct the observations at the three different stations. Good instruments were provided; one telescope having been procured by Dr. Franklin, at London, with the money voted by the Assembly. Another was sent by Thos. Penn, one of the proprietors, with the request, that after it had been used for the transit it should be given to the college. Other instruments were provided in sufficient number and of good quality. The long-looked-for moment was approaching. A large concourse of persons from the country, as well as the city, had crowded about the observatory, and there was some apprehension that the perfect silence necessary to the occasion might be broken. But an observer says, "so great was the anxiety of the company during the critical period, that there could not have been a more solemn pause of silence and expectation, if each individual had been waiting for the sentence that was to give them life or death." The observations at Philadelphia, Norriston, and Cape Henlopen, were all successful. In no part of the world were they more successful or more important.

No measure of public improvement was hazarded without seeking the counsel of this society, from the early project for the immense excavations which connect the Atlantic with the great Lakes of the West, to the best method of treating soils, and their adaptation to given products.

From 1774 there were frequent interruptions to the meetings of the society. No one acquainted with the history of the times need ask the reason. There may be duties sometimes required of us, as men and as citizens, before which, the pursuits of science, however useful or attractive, must give place. At the period of these interruptions, the encroachments of the British government occupied the thoughts and engaged the active exertions of all true patriots, to the exclusion of such pursuits as require leisure and a mind at ease. On a temporary resumption of the meetings, the following note appears in the handwriting of Dr. Rush.

"The acts of the British Parliament for shutting up the port of Boston, for altering the charter, and for the more impartial administration of justice in the province of Massachusetts Bay, having alarmed the whole American colonies, the members of the American Philosophical Society partaking with their countrymen in the distress and labors brought upon their country, were obliged to discontinue their meetings for some months, until a mode of opposition to the said acts of Parliament was established, which they hope will restore the former harmony, and maintain a perpetual union between Great Britain and her American colonies." Only a few months after this hope of harmony and perpetual union was expressed, the writer was himself one of the signers of that memorable instrument, which declared the eternal separation of these American colonies from the mother country.

It would be unjust to the many societies in this and foreign countries, to pass them by without a brief acknowledgment of their utility. They are mostly devoted to the elucidation of some particular department, and thus become mutual auxiliaries to those already more carefully described.

The association for the advancement of science in this country, has now a more vigorous existence than any other, although it has existed but a few years. Its meetings, which are held annually, are changed to different parts of the Union. Its productions have been greatly augmented during the last two years, and the scope of its labors extended.

The study of medicine in Paris is pursued in connection with the school of Medicine, which consists of twenty-six Professors, chosen by concours. This school like every other in France, is supported by Government, from which the Professors receive a fixed salary. They deliver their lectures in the Halls of the school of Medicine, and at the various Hospitals, as seem best to subserve the purposes of instruction. A student who proposes to receive the honors of the Paris school, must fulfill the following conditions. He must furnish a registry of age, and must not be under eighteen, must pursue his studies during four years, and every third month must inscribe his name at the bureau of the faculty. No note is taken of the way and place he spends his time in the intervals.

It is only required that he attain to a certain amount of knowledge in the given departments, which is proven by his examinations, which are held at stated times during his pupilage.

Thus, the student who has taken out five inscriptions is entitled at the beginning of his second year to an examination in *Chemistry*, *Natural History*, and *Botany*. At the end of three years he is examined in Anatomy and Physiology. The fifth and last examination is entirely practical, and based upon cases selected in one of the Hospitals, in which the candidate is expected to give the *diagnosis*, *prognosis*, and treatment.

In London and in this country the methods of instruction are nearly alike. The principal difference consists in the arrangement of medical schools connected with the Hospitals, which schools have no power to grant Degrees or Licenses.

The lectures are delivered within the walls of the hospital during winter sessions, but students may be in attendance at the hospital during the entire year.

Of the Boards to grant licenses or to confer degrees there are three, or two grades. The first is Apothecaries' Hall, to which Board the greatest number of general practitioners in Great Britain apply for their License to practice. The others are the Royal College of Physicians, and the Royal College of Surgeons, two distinct ordeals, and calculated to promote the more perfect acquisition of science, preparatory to practising in either department. Candidates from the several Hospitals of London, and elsewhere in the British dominions, who aspire to the highest honors conferred upon medical or surgical candidates, appear before these examiners, whose Diploma not only entitles one to practice, but admits them as members of these colleges.

I should not be acting in accordance with the impulses of my own feelings, if I should omit on this occasion to address myself immediately to those who intend to pursue their studies in these Halls.

Allow me to assure you with the earnestness of the deepest conviction, that your success and happiness are much more independent of the accidents and caprices of fortune, so called, that they are infinitely more within the sphere of your own control, than they appear to be, to the superficial observer. The avenues to honorable fame, in the profession of your choice, are widely open to you, or at least they are guarded by no barriers of which you may not command the key. In order to become successful practitioners in your chosen profession, you must become careful observers of the operations of nature, you must study her laws, and in the language of holy writ, "meditate upon them day and night." The time has undoubtedly gone by when the plea of being mere practitioners of any art shall serve as a cloak for indolence and ignorance.

The honors and emoluments that are due to the well informed practitioner of any art or science can in our day, only be acquired by diligent application and the most frugal use of time, which is not meted out to us in great measure. The point of time allotted to man, if occupied in its fullest measure, admits of but a brief and imperfect survey of the wonderful works of creation; the most beautiful though the most complicated of all, is the study of man.

You will find when you come to measure yourselves with men (and this you must surely do), that there are no rivals so formidable as those earnest determined minds, who reckon upon the value of every hour, and who achieve eminence, only by persistent application. Ambition for eminence in *literature* or *science* may influence you at certain periods, and the thought of some great names, will flash like a spark into the mine of your purpose.

You may dream till midnight over books, you may set up shadows and chase them down; other shadows, and they flee away—dreaming you never catch them. Nothing makes the scent lie well, in the hunt after honor and distinction, but application—persistent application.

We have the example of all who have been any credit to themselves or use to the world, that these are the only certain elements of success.

Let us then begin our studies for the session, not only with that ardor and zeal which becomes the student who is endeavoring to qualify himself for an honorable and useful profession, but with the recollection ever before us, that what we do in this life we must do quickly. Let us strive, in every relation to "walk circumspectly, not as fools, but as wise redeeming the time."

